# Associations between children's social functioning and physical activity participation are not mediated by social acceptance: a cross-sectional study

**DOI:** 10.1186/1479-5868-8-106

**Published:** 2011-09-30

**Authors:** Simon J Sebire, Russell Jago, Kenneth R Fox, Angie S Page, Rowan Brockman, Janice L Thompson

**Affiliations:** 1 Centre for Exercise Nutrition and Health Sciences, School for Policy Studies, University of Bristol, Bristol, BS8 1TZ, United Kingdom

**Keywords:** Social functioning, social acceptance, physical activity, accelerometer

## Abstract

**Background:**

Physical activity (PA) during childhood often occurs in social contexts. As such, children's ability to develop and maintain friendship groups may be important in understanding their PA. This paper investigates the associations among children's social functioning, and physical activity and whether perceptions of social acceptance mediate any social functioning-PA association.

**Methods:**

A cross sectional survey in which 652 10-11 year olds self-reported their peer (e.g. difficulties with friends) and conduct (e.g. anger/aggression) problems, prosocial behaviours (e.g. being kind to others) and perceptions of social acceptance. Physical activity was objectively assessed by Actigraph GT1M accelerometers to estimate counts per minute, (CPM) and minutes of moderate-to-vigorous physical activity (MVPA). Linear regression analyses were conducted to investigate associations between social functioning and PA. Indirect effects were analysed to explore mediation by social acceptance.

**Results:**

Among boys, peer problems were negatively associated with CPM and MVPA and conduct problems were positively associated with CPM and MVPA. Prosocial behaviour was unrelated to PA in boys. Social functioning was not associated with PA among girls. Social acceptance did not mediate the social functioning-PA relationship.

**Conclusions:**

Boys' conduct and peer problems were associated positively and negatively respectively with their PA but this relationship was not mediated by perceptions of social acceptance. Future research should study alternative mediators to understand the processes underpinning this relationship.

## Background

Physical activity (PA) is associated with improved mental well-being, lower levels of obesity and a reduced prevalence of cardiometabolic risk factors among children [1,2]. Many children in Western countries such as the UK and USA do not engage in sufficient amounts of PA to meet public health guidelines [3] and PA declines during childhood. As such, understanding the factors associated with children's PA behaviour, particularly before they make the transition to secondary school is a public health priority [4].

Most forms of PA during childhood such as play, and informal and organised sport/exercise [5-7] occur within a social context, with friends and friendship groups. For example, children play active games with their friends, take part in team sports/games both formally and informally in and out of school and just hang out with friends which may offer opportunities to be active (such as walking around town, going out on bikes). Accordingly, understanding factors supporting children's ability to develop and maintain friendship groups, such as their effective functioning in social contexts and perceptions of acceptance amongst their peers may advance our understanding of their PA participation.

There is accumulating evidence to suggest that children who have friends who are supportive and encouraging of their PA and offer opportunities to co-participate in PA may be more physically active than those who do not have such support systems [8-11]. Children's development and maintenance of a network of friends who can support their PA may be facilitated by the degree to which their socio-emotional and social-cognitive skills allow them to function effectively with their peers [12]. This hypothesis is supported by data showing that popular children (i.e., those most often rated by the members of their peer group as "liked") exhibit more developed social skills such as lower aggression, lower withdrawal and greater sociability than those less popular [13]. It has also been reported that adolescents who find it difficult to make friends report lower PA than those who find making friends less difficult [14]. Therefore, it can be hypothesised that how children get on with their friends and their functioning in friendship groups may be important in understanding how friendship dynamics influence their PA.

The construct of social functioning among children comprises active involvement in home life, interactions with family members and peers and the development and enactment of cognitive, physical and social skills and compliance with rules [15]. Children's social functioning is commonly measured using concepts of conduct problems (e.g., aggression & dishonesty), peer-problems (e.g., being isolated from friends) and prosocial behaviour (e.g., positive social actions) in addition to emotional symptoms and hyperactivity [15-17].

Children's strengths and difficulties (i.e., a composite of conduct problems, peer-problems, emotional symptoms and hyperactivity subscales) has been previously associated with lower PA levels [18]. Using a composite social functioning score however prevents examination of the associations between the individual components of social functioning and PA and previous research suggests that these individual components might be differently associated with PA. For example, peer problems may be more strongly related to children's PA due to their more direct link with the social context in which their activity takes place. Brodersen et al [19] reported negative cross-sectional associations between peer-problems and self-reported PA, a positive association between pro-social behaviour and PA among 11-12 year old boys and girls and a positive association between conduct problems and PA among boys but not girls. In contrast, Wiles, et al. [20] found that participation in sporting activities was unrelated to conduct problems, peer problems or pro-social behaviour reported one year later among 11-14 year old boys and girls. The primary aim of this study was to extend this literature by examining in greater detail the associations between individual social functioning subscales and children's PA.

A further limitation of the existing social functioning-PA literature is the use of subjective self-report or parent-report of PA or PA proxy measures (i.e., sport/exercise participation). While convenient and cost-effective in large-scale survey research, the limitations of self-reported PA measures (e.g., social desirability biases, recall errors and dishonesty) are well documented [21]. We sought to build on the previous literature by analysing the associations between individual social functioning components and objectively-assessed PA using accelerometers. Accelerometry provides more accurate estimates of the volume and intensity of PA activity at different intensities across the day and week.

Previous research is also confined to the examination of direct associations between social functioning and PA. However, recent calls have been made to understand in more detail the mediating mechanisms underpinning PA behavior [22] and it is therefore important to identify the potential mediators of any social functioning-PA relationship. Given the importance of friends in children's PA and associations between social functioning and popularity amongst peers [13] the construct of social acceptance (i.e., the perception of popularity/acceptance by one's peers) [23] may be a candidate mediator in the social functioning-PA relationship. Social acceptance is positively associated with sports participation [24], sports enjoyment, motivation and perceptions of competence [25] and self-reported physical activity [26] amongst youth. As children with more developed social skills experience greater popularity amongst their peers [13] children's social functioning may be associated with perceptions of social acceptance. Conduct and peer problems may undermine social inclusion and feelings of connectedness with peers [27] whereas pro-social behaviour may bolster interpersonal relationships and has been previously positively associated with peer acceptance [28].

**Figure 1:**
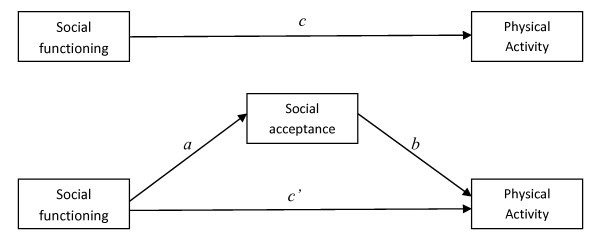
**Hypothesised mediation model**.

In summary, our primary aim was to examine the associations among children's social functioning and objectively measured PA. We hypothesised that peer problems and conduct problems would be negatively associated with PA as these are likely to undermine the social conditions that facilitate children's PA such as playing with friends or joining team games. We also hypothesised that pro-social behaviour would be positively associated with children's PA because it facilitates the social conditions for children's PA. As the association between social functioning components and PA may differ between boys and girls [19] we analysed associations among boys and girls separately. Our secondary aim was to test whether perceptions of social acceptance mediated any social functioning-PA relationship. We made the following mediation hypotheses; (a) peer problems and conduct problems would be negatively associated with PA because these factors would negatively predict children's perceptions of social acceptance and (b) pro-social behaviour would be positively associated with PA due to its positive association with social acceptance (see Figure 1).

## Methods

### Sampling and participants

A cross-sectional survey was conducted with data collected between April 2008 and March 2009. Data reported herein were collected within the larger 3Ps (Parents, Peers & Physical Activity) Project http://www.bris.ac.uk/enhs/research/recentprojects/bristol3ps.html. Ethical approval was granted by a University of Bristol ethics committee and informed parental consent was obtained for all participants. Data were collected from 40 primary schools in Bristol and participants were Year 6 children (10-11 years old). Primary schools were sampled based on the Index of Multiple Deprivation (IMD) for the school postcode. The IMD score estimates area deprivation based on indicators of income, health, educational and employment status [29]. Higher IMD scores indicate greater deprivation (i.e., lower socioeconomic position). IMD scores for all primary schools within 15 miles of the University of Bristol were obtained and schools were randomly selected from tertiles of IMD score. A total of 1684 Year 6 children were invited to participate and 1026 provided parental consent (60.9%); 986 pupils provided some data, with the remaining students absent during data collection. Of the 986 pupils, 652 (66%) participants provided complete social functioning, social acceptance, gender and sufficient accelerometer data and were used in analyses. Participants were 296 boys (*M*_age _= 10.91 years; *SD *= .40) and 356 girls (*M*_age _= 10.92 years; *SD *= .43). On average, data were collected from 17 children per school (range = 6 - 35).

### Measures

#### Social Functioning

Children's social functioning was measured using the prosocial, peer problems and conduct problems sub-scales of the Strengths and Difficulties Questionnaire (SDQ) [16,17]. The prosocial scale consists of 5 items assessing positive social actions (e.g., *I am kind to younger children*). The peer problems scale consists of 5 items assessing the degree to which the child experiences difficulties with their peers (e.g., *I am usually on my own. I generally play alone or keep to myself*). The conduct problems scale consists of 5 items assessing anger, aggression and dishonesty (e.g., *I get very angry and often lose my temper*). Participants indicated agreement using a 3-point likert-type scale; 0 (*Not true*), 1 (*Somewhat true*) and 2 (*Certainly true*). Subscale scores can range from 0 to 10. Higher prosocial subscale scores reflect more prosocial actions whereas higher peer and conduct problem scores indicate poorer social functioning. In the present study internal consistencies of the subscales were; prosocial α = .66, peer problems α = .60 and conduct problems α = .60. While these internal consistencies are below the commonly used .70 threshold [30] they are consistent with reliability coefficients obtained previously in cohorts of British children [17].

#### Social acceptance

The 6-item social acceptance subscale of Harter's Self-perception Profile for Children [23] was used to assess the degree to which children felt popular or accepted by their peers. Participants are presented with statements in a structured alternative format in which they are asked firstly to decide which of two statements (e.g., "*Some kids have a lot of friends" *and "*Other kids don't have very many friends"*) most closely describes them and then rate the chosen statement as either "*Sort of true for me" *or "*Very true for me"*. Average social acceptance scores can range from 1 to 4 and higher scores represent greater perceptions of social acceptance. In the present study the internal consistency (α = .65) was consistent with previous findings in British children [31]. All questionnaires were completed on handheld Personal Digital Assistant (PDA) devices in small groups (5 to 10 participants per group) supervised by a research assistant who addressed questions/difficulties.

#### Physical Activity

The ActiGraph GT1M accelerometer (Actigraph, Pensacola, Florida) was used to assess children's PA. Following the completion of questionnaires, participants were instructed to wear the accelerometer for five consecutive days including a weekend day on their hip during waking hours and to remove it when taking part in water-based activities and bathing. Acceleration was measured in 10 second epochs. 60 minutes of continuous zero counts was considered indicative of non-wear and these periods were removed from further analysis [32]. Days that consisted of ≥ 500 minutes of data were considered valid [33]. Participants who provided ≥ 3 valid days of data were included in the analysis [34]. Mean counts per minute (CPM) per day and after school (i.e., 3 pm to 6 pm) were determined to provide an estimate of PA volume. The after school period was selected as this is when children may have the best opportunity to be active with their friends and accrue much of their daily PA [35] and was calculated from weekday data only. Mean minutes of moderate-to-vigorous intensity PA (MVPA) per day and in the period after school was estimated using a cut-point of ≥ 3200 CPM [36]. As previous research suggests that values obtained from the GT1M are 9% higher than the values obtained from the accelerometer model (Actigraph 7146) employed in deriving the threshold [37] a correction factor of 0.91 was applied to this threshold to yield an MVPA cut point of 2912 CPM.

### Data Analysis

Descriptive statistics were calculated for all variables and independent samples *t*-tests were used to examine mean differences between boys and girls. Relationships among variables were examined using bivariate correlations. Linear regression analyses were conducted to examine the prediction of variance in PA variables by SDQ social functioning scores. Statistical assumptions of regression analyses (i.e., linearity, homoscedasticity and independence and normality of residuals) were tested [38].

To test mediation models we performed a series of regression analyses and examined indirect effects [39,40]. This procedure involves calculating the following (Figure 1): (a) the effect of the predictor variable (SDQ variable) on the PA outcome variable (path *c*); (b) the effect of the predictor on the (social acceptance) mediator (path *a*); (c) the effect of the mediator on the outcome variable controlling for the predictor (path *b*); (d) the indirect effect from the predictor to the outcome via the mediator (i.e., a*b); and (e) the direct effect of the predictor variable on the outcome controlling for the mediator (*c'*). The indirect effect is determined by examining bootstrapped and bias-corrected confidence intervals [41]. Bootstrapping is a re-sampling technique in which a statistic (e.g., the indirect effect) is estimated in multiple same-sized samples drawn from the original sample with replacement (i.e., participant 1 can be randomly selected into the first bootstrapped sample, replaced back into the original participant pool and randomly selected again). The distribution of these estimates is analysed and a confidence interval around a point estimate of the indirect effect is created. In the present analysis 5000 bootstrap samples of the same size as the original sample with replacement were requested. IMD score, hours of daylight on the first day of data collection and all SDQ variables were entered as covariates.

Given that gender differences in the association between social functioning and PA have been previously reported [19] analyses were conducted separately for boys and girls. Data were analysed using Stata version 9.0 (College Station, Texas). Robust standard errors were examined to account for clustering of children within schools. Mediation analysis was performed using an in-house Stata programme.

## Results

### Preliminary results

Descriptive statistics are reported in Table 1. SDQ conduct and prosocial scores were similar to gender-specific norms for British children of similar age and peer problems scores were marginally higher http://www.sdqinfo.com. Independent sample *t*-tests revealed that peer problem scores did not differ significantly between genders, whereas girls reported significantly fewer conduct problems and significantly greater prosocial behaviour than boys. Social acceptance scores were moderate to high and did not differ significantly between boys and girls. Boys engaged in significantly greater PA (CPM and MVPA) daily and after school than girls. Boys performed approximately 42 minutes of MVPA per day, 12 minutes of which were performed after school. Girls performed approximately 30 minutes of MVPA, 9 minutes of which were performed after school.

**Table 1:** Participant characteristics, descriptive statistics and contrasts between 296 boys and 356 girls.

	Total sample		Boys		Girls				
				
Variable	*M*	*SD*	*M*	*SD*	*M*	*SD*	*t*(df)	*p*	*Hedges' g*
Age (years)	10.92	.42	10.92	.40	10.91	.43	-.23 (609)	.82	-.02
SDQ^1 ^- Conduct problems	2.25	1.70	2.45	1.73	2.07	1.66	-2.85 (650)	.01	-.22
SDQ - Peer problems	1.91	1.74	1.97	1.83	1.88	1.69	-.61 (650)	.54	-.05
SDQ - Prosocial	7.99	1.70	7.52	1.78	8.38	1.54	6.50 (586.38^4^)	.00	.52
Social acceptance	3.05	.64	3.05	.65	3.06	.64	.18 (650)	.84	.02
Mean MVPA^2 ^per day (min)	35.82	17.43	42.23	19.22	30.28	13.30	-9.08 (509.60^4^)	.00	-.73
Mean CPM^3 ^per day	543.75	168.07	592.27	173.22	501.56	150.66	-7.06 (589.13^4^)	.00	-.56
Mean MVPA after school per day (min)	10.13	6.65	11.63	7.54	8.82	5.46	-5.32 (516.44^4^)	.00	-.43
Mean CPM after school per day	663.33	322.68	700.40	329.49	630.98	314.17	-2.71 (633)	.01	-.22

^1^SDQ = Strengths and Difficulties Questionnaire.

^2 ^MVPA = moderate-to-vigorous physical activity.

^3 ^CPM = counts per minute.

^4 ^Scatterthwaite's approximation of degrees of freedom based on unequal variances.

† = *p *< .10, * = *p *< .05, ** = *p *< .01.

Bivariate correlations are presented in Table 2. Conduct problems were not associated with daily MVPA or MVPA after school in either boys or girls. Peer problems were negatively associated with full day MVPA, MVPA after school and CPM after school among boys, suggesting fewer peer problems in the more active boys. Peer problems were not associated with any PA measure among girls. Prosocial behaviour displayed a negative association with full day MVPA among girls but was not associated with boys' PA. Social acceptance scores were significantly negatively associated with SDQ conduct and peer problem scores and were positively correlated with prosocial behaviour among boys and girls, therefore providing support for possible mediation. Further, feelings of social acceptance were positively correlated with full day MVPA, MVPA after school and CPM after school in boys and displayed a marginally significant positive correlation with MVPA among girls.

**Table 2:** Bivariate correlations among study variables in boys (top) and girls (bottom).

		1	2	3	4	5	6	7	8	9	10
1	IMD^1 ^Score	1									
		1									
2	Daylight	.24**	1								
		.38**	1								
3	SDQ^2 ^Conduct problems	-.07	.01	1							
		-.04	-.10†	1							
4	SDQ Prosocial	.04	.10†	-.30**	1						
		.02	.01	-.38**	1						
5	SDQ Peer problems	-.07	-.01	.31**	-.11†	1					
		-.14*	-.11*	.27**	-.05	1					
6	Social acceptance	.11†	.07	-.20**	.13*	-.50**	1				
		.18**	.09	-.16**	.20**	-.47**	1				
7	Mean MVPA^3 ^per day (min)	.12*	.19**	.10†	-.03	-.13*	.12*	1			
		.13*	.21**	.09	-.10*	-.06	.09†	1			
2	Mean CPM^4^	.07	.33**	.11†	.01	-.11†	.11†	.72**	1		
		.10†	.27**	.08	-.06	.00	.04	.62**	1		
9	Mean MVPA after school (min)	.15*	.08	.04	-.04	-.16*	.12*	.58**	.60**	1	
		.06	.00	.04	.10†	.01	.02	.63**	.59**	1	
10	Mean CPM after school	.11†	.27**	.07	.06	-.17*	.16*	.53**	.76**	.79**	1
		.05	.11*	.05	-.07	.07	-.04	.44**	.79**	.74**	1

^1 ^*IMD = *Index of Multiple Deprivation.

^2 ^SDQ = Strengths and Difficulties Questionnaire.

^3 ^MVPA = moderate-to-vigorous physical activity.

^4 ^CPM = counts per minute.

† = *p *< .10, * = *p *< .05, ** = *p *< .01

### Primary results

Screening of regression assumptions revealed no violations. Table 3 presents the regression results. Among boys, after controlling for IMD score, hours of daylight and the remaining SDQ variables, conduct problems were positively associated with MVPA, CPM and CPM after school. Peer problems were negatively associated with all PA variables. Prosocial behaviour was not associated with PA among boys. The variance explained in boys' PA by social functioning ranged from 6% to 13%. Among girls, the regression analysis revealed that none of the social functioning variables predicted their PA. Analyses were repeated using weekend MVPA and CPM as outcomes and no significant associations were identified with social functioning or social acceptance.

**Table 3:** Linear regression models predicting physical activity from social functioning in boys and girls.

	Boys			Girls		
	
Outcome variable = MVPA^6 ^per day						
	
Variable	B^1^	SE^2^	95% CI^3^	B	SE	95% CI
IMD^4 ^score	.08	.08	[-.07, .24]	.04	.04	[-.03, .12]
Daylight	.00**	.00	[.00, .00]	.00**	.00	[.00, .00]
SDQ^5 ^Conduct problems	1.63*	.69	[.23, 3.03]	.83	.53	[-.26, 1.91]
SDQ Prosocial	-.38	.71	[-1.82, 1.10]	-.42	.34	[-1.11, .27]
SDQ Peer problems	-1.79*	.68	[-3.18, -.40]	-.55	.46	[-1.48, .38]
	R^2 ^for model = .09			R^2 ^for model = .06		

Outcome variable = CPM^7 ^per day						
	
Variable	B	SE	95% CI	B	SE	95% CI

IMD score	-.13	.56	[-1.26, 1.01]	-.00	.52	[-1.05, 1.05]
Daylight	.01*	.00	[.00, .01]	.01**	.00	[.00, .01]
SDQ Conduct problems	14.23**	4.11	[5.91, 22.56]	9.85†	5.57	[-1.43, 21.14]
SDQ Prosocial	-1.73	5.90	[13.69, 10.24]	-.23	4.91	[-10.18, 9.72]
SDQ Peer problems	-15.44**	5.56	[-26.70, 4.18]	-.91	5.10	[-11.22, 9.41]
	R^2 ^for model = .13			R^2 ^for model = .08		

Outcome variable = MVPA after school						
	
Variable	B	SE	95% CI	B	SE	95% CI

IMD score	.06*	.03	[.00, .11]	.02	.02	[-.01, .05]
Daylight	.00	.00	[-.00, .00]	-.00	.00	[-.00, .00]
SDQ Conduct problems	.30	.22	[-.15, .75]	.11	.21	[-.32, .55]
SDQ Prosocial	-.26	.23	[-.75, .22]	-.15	.17	[-.51, .21]
SDQ Peer problems	-.79**	.24	[-1.26, -.31]	-.06	.20	[-.47, .35]
	R^2 ^for model = .06			R^2 ^for model = .01		

Outcome variable = CPM after school						
	
Variable	B	SE	95% CI	B	SE	95% CI

IMD score	.80	1.09	[-1.41, 3.00]	.32	1.03	[-1.78, 2.41]
Daylight	.01**	.00	[.00, .01]	.00	.00	[-.00, .01]
SDQ Conduct problems	24.41**	9.58	[4.99, 43.82]	5.14	10.43	[-15.99, 26.28]
SDQ Prosocial	8.07	9.53	[-11.25, 27.38	-7.80	9.33	[-26.72, 11.12]
SDQ Peer problems	-36.61**	9.31	[-55.48, -17.74]	10.62	10.70	[-11.06, 32.30]
	R^2 ^for model = .11			R^2 ^for model = .02		

^1^B = unstandardized beta coefficient.

^2^SE = robust standard error.

^3^CI = confidence interval.

^4^IMD *= *Index of Multiple Deprivation.

^5^SDQ = Strengths and Difficulties Questionnaire.

^6 ^MVPA = moderate-to-vigorous physical activity.

^7 ^CPM = counts per minute.

† = *p *< .10, * = *p *< .05, ** = *p *< .01

Given statistically significant bivariate correlations among the variables specified in the hypothesised mediation models and that analysis of mediation through indirect effects can continue in the absence of an initial direct effect (e.g, a significant association between social functioning and PA, path *c*, Figure 1) [39] mediation analysis was pursued.

Among boys, regression analysis revealed that neither conduct problems nor prosocial scores were associated with the social acceptance mediator (path *a*, Figure 1) thus preventing social acceptance acting as a mediator of the effects of these variables. For peer problems a significant negative association (*b *= -.17, *p *= ≤ .01) with social acceptance was found (path *a*, Figure 1). However, the associations between social acceptance and the PA variables (path *b*, Figure 1) was not significant, suggesting that social acceptance did not mediate the peer problems-PA relationship among boys.

Among girls, conduct problems were not associated with social acceptance (path *a*, Figure 1) thus preventing further mediation analysis using conduct problems. A small positive association (*b *= .07, *p *≤ .01) was identified between prosocial scores and social acceptance which was in turn weakly associated with full day MVPA (*b *= 1.86, *p *= ≤ .10) suggesting that mediation could exist. However, the indirect effect was not statistically significant (*b *= .14, bootstrapped standard error = .11, bootstrapped bias-corrected 95% confidence interval = -.01 to .42) ruling out mediation. For peer problems, a significant negative association was identified between peer problems and social acceptance (*b *= -.18, *p *≤ .01) which was in turn marginally significantly associated with MVPA per day (*b *= 1.86, *p*= ≤ .10). However, the indirect effect was not statistically significant (*b *= -.33, bootstrapped standard error = .21, bootstrapped bias-corrected 95% confidence interval = -.77 to .06) again ruling out mediation via social acceptance.

## Discussion

In the present study we identified cross-sectional associations between components of boys' social functioning and their PA assessed by accelerometer. A positive association was also found between social acceptance and PA among boys. We did not find support for the mediation of the social-functioning-PA relationship by perceptions of social acceptance. Consistent with our hypothesis, amongst boys, peer problems were negatively associated with accelerometer-derived measures of PA volume and MVPA per day and after school. Similar relationships have been identified among girls [19] but we did not replicate this finding. Boys and girls did not differ significantly in the level of peer problems they reported in this study. The lack of association identified among girls suggests that their perceived peer problems did not relate to their level of PA.

Consistent with previous research [19] we identified a positive association between conduct problems and PA among boys but not among girls. The finding among boys initially appears counterintuitive, however it has been previously suggested that boys exhibiting conduct problems (e.g., fighting, stealing, disobeying adults) may use or be encouraged to use PA to channel their aggression [19]. Conduct problems may also be associated with other behavioural disorders such as Attention Deficit and Hyperactivity Disorder which may also lead to greater PA [42]. An alternative explanation lies in the way conduct problems are conceptualised. In the SDQ, conduct problems are conceived mainly as aspects of children's behavior associated with adults (e.g., being disobedient). Such problems may not be an issue for other children (as indicated by a smaller negative correlation between conduct problems and peer acceptance than peer problems and peer acceptance) and could be seen as a marker of respect within some peer groups, facilitate group membership and opportunities to be active. Girls reported significantly lower conduct problems than boys and the lack of association between their conduct problems and PA may indicate that girls' conduct problems may be manifested in behaviours unrelated to PA. In contrast, boys conduct problems may be manifested in more active behaviours (i.e., spending more time outside of the home). In contrast to our hypothesis, prosocial behaviour was not associated with PA among boys or girls. Previous research has identified small positive associations between prosocial behaviour and self-reported PA [19]. One explanation for our different findings may be due to our objective measure of PA. As both PA and prosocial behaviours are socially desirable actions, previous associations identified between these variables based on self-reported data may be inflated due to a form of common method variance [43] underpinned by socially desirable responses. It is possible that this also explains why our findings for girls are different to previous research as girls may be more likely to provide socially desirable SDQ and PA responses. Alternatively, our measure of PA does not allow for a distinction between solitary PA and PA with other children. Prosocial behaviour may be more predictive of time spent being physically active with other children and our more general measure of PA may have masked any such associations within the data.

Although social acceptance was positively associated with both social functioning and PA, mediation analysis revealed that social acceptance did not mediate the social functioning-PA relationship. To advance understanding of the mechanisms underpinning associations between social functioning and PA, future research should seek to examine the role of other possible mediators of this relationship. Identifying mediating mechanisms is important for identifying targets for interventions to increase PA [22]. Previous research suggests that peer-based variables such as co-participation, support and encouragement influence children's PA [44] and that the association between social acceptance and PA may itself be mediated by variables more proximal to PA [45]. It is therefore plausible that social functioning may be associated with these variables. It is also possible that the association between different components of social functioning and PA could be mediated by different variables. For example peer problems may be associated with PA through a possible relationship with the number of friends a child has and this should be investigated alongside other potential mediators.

### Limitations & Future Directions

Our cross-sectional data does not provide evidence for the direction of causality amongst the variables. While we conceptualised social functioning as a precursor of PA, in line with previous research, it is entirely possible that engaging in PA (e.g., a team sport or team-based active pursuit) may have positive effects on a young person's social functioning or that effects are reciprocal.

The internal consistency reliability of the self-reported measures was low and similar problems have been reported previously [17,31]. The low reliability may have attenuated correlations between the variables [46] which may explain some of the null findings. In addition it is possible that boys and girls may provide different socially desirable responses to the SDQ which may partially explain the different findings between boys and girls. Parent and teacher-completed versions of the SDQ are available http://www.sdqinfo.com and future work should employ these alternative measures of social functioning. Previous work particularly supports the internal consistency of teacher-rated SDQ scores [17].

Our objective measure of PA was a strength of the study, however accelerometers are unable to measure participation in activities such as water sports and may have therefore underestimated PA among children who participate in such activities. Further, our PA measure did not capture the context of PA (i.e., playing outdoors or with friends) which may be particularly important when considering the effect of social functioning problems on PA of children and young people. Finally, our sample was drawn from one English city and the generalisability of our findings to children in other areas of the UK and internationally is limited. Future cross-cultural research exploring associations between social functioning and objectively measured PA is warranted as is longitudinal research to examine if the social functioning predicts future PA.

Studying peer influences on the PA of children in transition periods has been previously forwarded as an important route for future research [7] particularly as this transition coincides with decreases in adolescent PA [3]. In line with this suggestion, further research may build on the present study to investigate associations between social functioning, peer variables (e.g., best friend analysis) and PA over the transition from primary to secondary school. Finally, future research exploring the influence of social functioning and PA should seek to measure the time that children spend being physically active with other children, as it is these settings in which children's social functioning may be most salient.

## Conclusions

In the present study, boys' peer problems were associated with lower objectively-assessed daily PA and PA after school whereas greater conduct problems were associated with greater PA. Social functioning variables were unrelated to PA among girls. Perceptions of social acceptance did not mediate the social functioning-PA association among boys and further research testing alternative mediating mechanisms is warranted. Current public health policies focus on increasing the PA participation of young people [4]. This research suggests that minimising peer problems may be a potential strategy to include and test in future interventions to increase PA in boys.

## Competing interests

The authors declare that they have no competing interests.

## Authors' contributions

The project was conceived by RJ and the paper was conceived by SJS, RJ & KRF. All data were collected by RB. Analysis was performed by SJS. SJS led the drafting of the manuscript with all authors adding sections for the paper. All authors made critical contributions to the manuscript and approved the final version.
